# Gene Dysregulation and Islet Changes in PDAC-Associated Type 3c Diabetes

**DOI:** 10.3390/ijms26073191

**Published:** 2025-03-29

**Authors:** Jessica L. E. Hill, Eliot Leonard, Dominique Parslow, David J. Hill

**Affiliations:** 1 Keele Medical School, University of Keele, Newcastle ST5 5BG, UK; y0w14@students.keele.ac.uk; 2 Leeds Teaching Hospitals NHS Trust, Leeds LS1 3EX, UK; eliot.leonard1@nhs.net; 3 University Hospitals Plymouth, Plymouth PL6 8DH, UK; dominiqueparslow@nhs.net; 4 Lawson Research Institute, St. Joseph Health Care, London, ON N6A 4V2, Canada; 5 Departments of Medicine, Physiology and Pharmacology, Western University, London, ON N6A 3K7, Canada

**Keywords:** pancreatic ductal adenocarcinoma, type 3 diabetes, PD-L1, INS, islet basement membrane

## Abstract

Pancreatic ductal adenocarcinoma (PDAC) is a highly lethal malignancy, often associated with new-onset diabetes. The relationship between PDAC and diabetes, particularly type 3c diabetes, remains poorly understood. This study investigates whether PDAC-associated diabetes represents a distinct subtype by integrating transcriptomic and histological analyses. Whole-tumour RNA sequencing data from The Cancer Genome Atlas (TCGA) were analysed to compare gene expression profiles between PDAC patients with and without diabetes. Cell-type Identification By Estimating Relative Subsets Of RNA Transcripts (CIBERSORT) deconvolution was employed to assess immune cell populations. Histopathological evaluations of pancreatic tissues were conducted to assess fibrosis and islet morphology. Histological analysis revealed perivascular fibrosis and islet basement membrane thickening in both PDAC cohorts. Transcriptomic data indicated significant downregulation of islet hormone genes insulin (INS) and glucagon (GCG) but not somatostatin (SST) in PDAC-associated diabetes, consistent with a type 3c diabetes phenotype. Contrary to previous reports, no distinct immunogenic signature was identified in PDAC with diabetes, as key immune checkpoint genes (Programmed Cell Death Protein 1 (PDCD1), Cytotoxic T-Lymphocyte Associated Protein 4 (CTLA4), Programmed Death-Ligand 1(PD-L1)) were not differentially expressed. The findings suggest that PDAC-associated diabetes arises through neoplastic alterations in islet physiology rather than immune-mediated mechanisms. The observed reductions in endocrine markers reinforce the concept of PDAC-driven β-cell dysfunction as a potential early indicator of malignancy. Given the poor response of PDAC to PD-L1 checkpoint inhibitors, further research is needed to elucidate alternative therapeutic strategies targeting tumour–islet interactions.

## 1. Introduction

Pancreatic ductal adenocarcinoma (PDAC) is one of the most lethal malignancies, with a five-year survival rate of less than 10% and limited effective treatment options [1,2]. In a recent Phase II trial by O’Reilly et al. [3], the addition of durvalumab (anti–PD-L1) with or without tremelimumab (anti–CTLA-4) to standard chemotherapy conferred no significant improvement in outcomes for patients with metastatic PDAC. This finding has spurred heightened interest in delineating PDAC subtypes to inform tailored therapeutic strategies and in exploring diabetes as a potentially crucial early biomarker of PDAC, although the mechanistic links between PDAC and diabetes remain poorly understood. Estimates suggest that between 45% and 65% of patients diagnosed with PDAC have concurrent diabetes [4]. In contrast, diabetes is significantly less prevalent among individuals with non-pancreatic malignancies or age-matched controls [5,6]. Notably, hyperglycemia can precede a PDAC diagnosis by up to 36 months, offering a potential window for early cancer detection through pre-diabetes screening [7].

While previous studies have leveraged The Cancer Genome Atlas (TCGA) to characterize the PDAC transcriptome, many have relied on broad, unspecific analyses that incorporate mixed pancreatic pathologies that confound their findings. A potential limitation of certain earlier investigations, such as those by Yan et al. [8], Wu et al. [9], Zhou et al. [10], and Hu et al. [11], is suboptimal sample selection, leading to heterogeneous cohorts that inadvertently include non-PDAC pancreatic pathologies as well as patients with pancreatitis, a condition strongly associated with an inflammatory environment, secondary (type 3c) diabetes, and an increased malignancy risk [12].

Our study addresses these gaps by employing a rigorously curated TCGA dataset, ensuring that only histologically confirmed PDAC samples were included, thereby eliminating confounding pancreatic lesions and excluding patient samples with conditions such as pancreatitis. Moreover, rather than conducting an undirected immune profiling approach, we took a targeted strategy, focusing on immune checkpoint proteins—particularly PD-L1 and PD-1—which have been implicated in immunotherapeutic resistance in PDAC. This strategic decision was informed by prior studies, including Bailey et al. [13] and Yan et al. [8]. The latter indicated that PDAC-associated diabetes may be a distinct subtype of PDAC driven predominantly by immune mechanisms, suggesting that type 3c diabetes could render patients more sensitive to targeted immunotherapies (e.g., anti-PD-L1) compared to PDAC patients without diabetes. If confirmed, this would indicate a potential opportunity to treat patients with PDAC and diabetes specifically. In contrast, Gardi et al. [14] highlighted the complexity of metabolic reprogramming in PDAC, suggesting that diabetes-related molecular changes may not be uniformly immune-mediated. We chose to integrate transcriptomic analysis with histological assessments to provide a more precise characterization of immune phenotypes in PDAC with diabetes and to shed light on the potential limitations of immune-based therapeutic strategies alone.

The clinical implications of PD-L1 expression in PDAC cannot be overstated. While immune checkpoint blockade has revolutionized cancer therapy in other malignancies, PDAC remains largely refractory to these interventions. Understanding the nuanced expression of PD-L1 and its relationship to tumour–immune interactions is critical for refining patient selection criteria for immunotherapy.

Despite the significant focus on PDAC’s exocrine pathology, islet architecture and endocrine function have been largely ignored. This is likely due to the assumption that endocrine alterations are of secondary importance compared to the rapidly progressing neoplasm. Furthermore, the basement membrane (BM) of islets plays a crucial role in maintaining tissue integrity and regulating cell behaviour, yet its contribution to PDAC progression is poorly understood. The BM, composed of extracellular matrix proteins such as laminins and collagen IV, provides structural support and mediates cell signalling [15]. Disruption of the peri-islet BM has been implicated in islet dysfunction and immune evasion [16].

By adopting a selective, hypothesis-driven approach, this study provides a critical reassessment of immune checkpoint expression in PDAC-associated diabetes while contextualizing its findings within the broader landscape of pancreatic cancer research.

## 2. Results

### 2.1. Patient Data Collection and Curation

**Figure 1:**
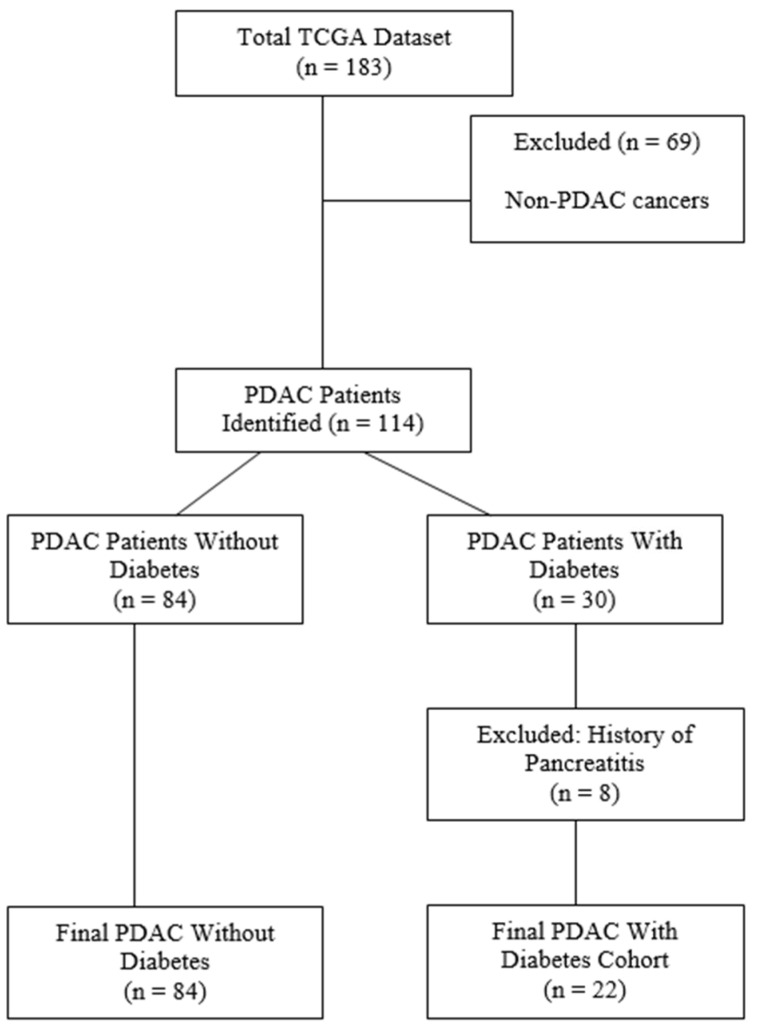
The selection process for the final study cohort. The total TCGA dataset initially included 183 samples, of which 69 were excluded due to non-PDAC cancers. A total of 114 PDAC patients were identified, which were further stratified into two groups: PDAC patients without diabetes (n = 84) and PDAC patients with diabetes (n = 30). Among the latter group, 8 patients with a history of pancreatitis were excluded, resulting in the final PDAC with diabetes cohort (n = 22). The final PDAC without diabetes cohort included 84 patients.

Comprehensive genomic and clinical data for PDAC were retrieved from TCGA and curated for accuracy. The final cohort consisted of 114 patients, including 22 with PDAC and diabetes and 84 without diabetes, after excluding non-PDAC cases and patients with a history of chronic pancreatitis (Figure 1). Quality control ensured the dataset included RSEM-normalized RNA-sequencing data and detailed metadata, enabling transcriptomic and clinical analyses.

### 2.2. Fibrosis Observed Within and Around the Islets of Langerhans

**Figure 2:**
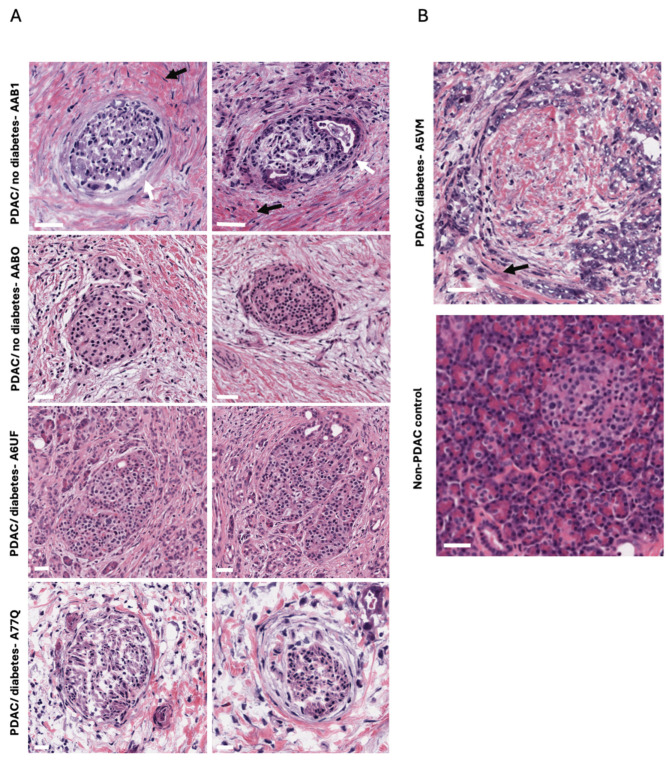
Islet pathology in PDAC. (**A**) Islets from individuals with PDAC are situated within tissue showing high levels of fibrosis (indicated by black arrow) and some demonstrate thickened basal membranes (indicated by white arrow). Representative islets are shown from the patients with no diabetes (AAB0 and AAB1) and those with diabetes (A77Q, A6UF). All samples were taken from PDAC tumours graded stage II (**B**) and located at the head of the pancreas. Pancreatic tissue from an individual with PDAC and diabetes showing high levels of fibrosis and alteration of the exocrine tissue (indicated by black arrow). Pancreatic tissue from non-PDAC/non-diabetic patient is shown for comparison. The scale bar represents 20 µm.

Examination of pancreatic tissue sections from the TCGA database revealed marked fibrosis and collagen deposition both within and around the islets of Langerhans in donors with PDAC. This peri-islet fibrotic reaction often replaced the normal acinar architecture, suggesting an extensive desmoplastic response consistent with PDAC pathology (Figure 2). In addition, in certain samples (e.g., non-diabetic AAB1 and diabetic A77Q), the islet basement membrane appeared notably thickened, although the overall internal architecture of the islets was largely preserved.

Initially, we identified three samples in the TCGA database (TCGA-H6-8124-11A, TCGA-PZ-A5RE-11A, TCGA-L1-A7W4-11A) described as “normal” pancreatic tissue; however, a closer review of both the histology and accompanying pathology reports confirmed that these tissues originated from patients with PDAC (TCGA-H6-8121-01A, TCGA-PZ-A5RE-11A, TCGA-L1-A7W4-01A). Consequently, no truly normal, non-PDAC control tissue was available within the same dataset, making direct comparisons with healthy islets impossible. To circumvent this limitation, we consulted the University of Exeter’s Diabetes Archival Database, which provides a large repository of pancreatic tissue from donors without PDAC and with diverse diabetes etiologies. These tissues serve as a reference for baseline islet architecture, basement-membrane thickness, and fibrotic content.

### 2.3. Endocrine and Progenitor Cell Markers Are Significantly Reduced in PDAC with Diabetes

**Figure 3:**
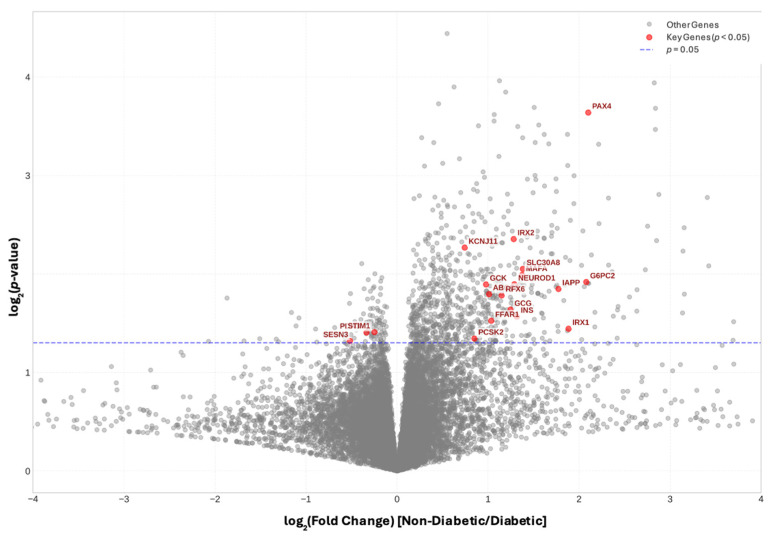
Volcano Plot of Differential Gene Expression in PDAC associated Diabetes vs. Non-Diabetes. Volcano plot showing the relationship between gene expression changes and statistical significance in PDAC patients with diabetes compared to those without diabetes. The horizontal blue dashed line indicates the significance threshold (*p* = 0.05). Grey dots represent individual genes, while red dots highlight key islet-related genes (*p* < 0.05) involved in glucose homeostasis and pancreatic function.

We first examined the expression of INS, GCG, and SST, which encode the primary islet hormones insulin, glucagon, and somatostatin, respectively. Notably, INS and GCG, levels were significantly decreased in donors with PDAC-associated diabetes compared to those with PDAC but no diabetes (Figure 3). GCK, which encodes the β-cell glucose sensor glucokinase [17], was also downregulated in the diabetes cohort, as was the glucagon receptor which allows glucagon to positively influence insulin release within the paracrine environment of the islet (Supplementary Table S1). We also observed a significant downregulation of several key proteins involved in β-cell function and insulin secretion. These included Potassium Two Pore Domain Channel Subfamily K Member 16 (KCNK16), a potassium channel regulating β-cell membrane potential; Double C2 Domain Alpha (DOC2A) and Regulating Synaptic Membrane Exocytosis 2 (RIMS2), both critical for insulin granule docking and exocytosis; BAI1 Associated Protein 3 (BAIAP3), which governs vesicle trafficking; and Chromogranin A (CHGA), a core component of secretory granules in endocrine cells. A number of G-protein coupled receptors (GPCRs) allowing peptide and nutrient regulated modulation of insulin release were also reduced in expression (Supplementary Table S1). Of the major islet hormones only expression of SST was not significantly reduced in diabetes-associated PDAC, although both Somatostatin Receptor 3 (SSTR3) and SSTR5 which allow paracrine signaling of somatostatin to surrounding α- and β-cells were decreased. MAF BZIP Transcription Factor A (MAFA), Neuronal Differentiation 1 (NEUROD1) and NEUROD4 were significantly downregulated (*p* < 0.05) in the diabetes cohort, suggesting a disruption in the transcriptional regulation of β-cell identity and function. A number of other transcription factors associated with islet endocrine cell lineage commitment were also downregulated, such as Paired Box 4 (PAX4), Homeobox B Cluster (HOXB) genes, Nanog Homeobox (NANOG) and NK1 Homeobox 2 (NKX1-2) (Supplementary Table S1). Similarly, a number of peptide growth factors and/or their receptors known to mediate their ligand actions on islet cell replication and survival also showed lower expression, including Platelet Derived Growth Factor Subunit A (PDGFA), Fibroblast Growth Factor Receptor 4 (FGFR4) and Hepatocyte Growth Factor Activator (HGFAC). Other genes downregulated were associated with fibrosis and modification of extracellular matrix such as SMAD Family Member 3 (SMAD3), Disintegrin And Metalloproteinase Domain-Containing Protein 32 (ADAM32) and C-X-C Motif Chemokine Ligand 5 (CXCL5), and their disruption may be associated with the islet-associated fibrosis observed in PDAC.

Interestingly, despite the extensive gene expression alterations, no distinct immunogenic signature was identified, as immune checkpoint genes such as CD274 (PD-L1), PDCD1 (PD-1), and CTLA4 did not show significant differential expression between the two cohorts.

**Figure 4:**
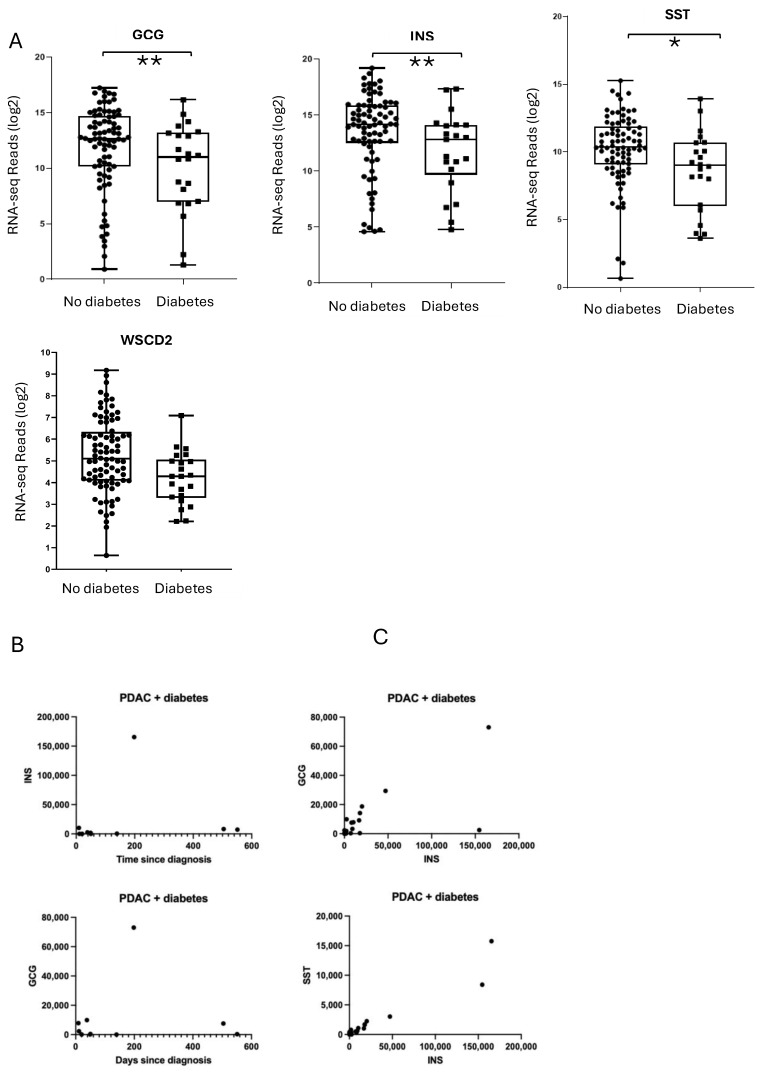
Gene expression analysis of endocrine markers across cohorts. (**A**) Endocrine markers INS, GCG, SST, and WSCD2 were all reduced in the diabetic cohort (** *p* < 0.05, * *p* < 0.1) (**B**) Correlation analysis between duration of diabetes (days) and INS (r = 0.36); GCG (r = −0.06). (**C**) Correlation analysis between GCG and INS (r = 0.79; *p* < 0.001), and SST and INS (r = 0.84; *p* < 0.001) for the diabetic cohort. Gene expression levels are represented as RSEM-normalized counts.

To determine whether these gene expression reductions reflect a global islet deficit rather than a selective depletion of specific islet cell subtypes, we performed correlation analyses. In PDAC donors with diabetes, INS positively correlated with both GCG and SST (Figure 4), suggesting a simultaneous loss of multiple endocrine cell types. Notably, disease duration (i.e., days since diabetes diagnosis) did not correlate with the magnitude of endocrine gene downregulation; several long-duration diabetic donors retained moderate hormone gene expression, whereas others with shorter disease duration had very low levels (Figure 4B). This variability is likely influenced by sampling differences in pancreatic tissue damage and tumour infiltration.

We also observed positive correlations between WSC Domain Containing 2 (WSCD2) and both INS (r = 0.56, *p* = 0.006) and GCG expression (r = 0.52, *p* = 0.01). WSCD2 is enriched in β-cells compared to α-cells or exocrine tissue [18] and has been shown to decrease under diabetic or hyperglycemic conditions, paralleling insulin loss. These findings reinforce the notion that PDAC-associated diabetes affects the entire endocrine compartment of the pancreas rather than selectively impairing one islet cell lineage.

As shown in Supplementary Figure S1, transcripts encoding the β-cell transcription factors MAFA and PAX4 mirrored the reductions in INS. In particular, MAFA was significantly downregulated in the diabetic group and closely correlated with INS expression. Consistent with rodent knockout studies [19,20], the correlation between GCG and PAX4 in diabetic donors suggests that reduced PAX4 may contribute to the global endocrine phenotype rather than selectively influencing α- or β-cell fate. Expression of Aristaless Related Homeobox (ARX) and MAFB, which are typically associated with mature α cells, did not correlate with GCG in the diabetic group, although these markers showed strong associations in the non-diabetic donors (*p* < 0.001). This pattern further supports a more global disruption of islet maintenance programs in the PDAC-associated diabetes cohort.

Several transcription factors linked with β-cell neogenesis, and cellular metabolic and immune stress were upregulated in PDAC-associated diabetes, including Recombination Signal Binding Protein For Immunoglobulin Kappa J Region (RBPJ), Aryl Hydrocarbon Receptor Nuclear Translocator (ARNT) and Forkhead Box P1 (FOXP1), as well as a pro-apoptotic gene, BCL2 Like 11 (BCL2L11) (Supplementary Table S1). Increased expression of Cartilage Associated Protein (CRTAP)which is associated with collagen formation may be associated with the observed islet fibrosis.

### 2.4. Exocrine Gene Expression Profiles Are Comparable Between PDAC Donors with and Without Diabetes

**Figure 5:**
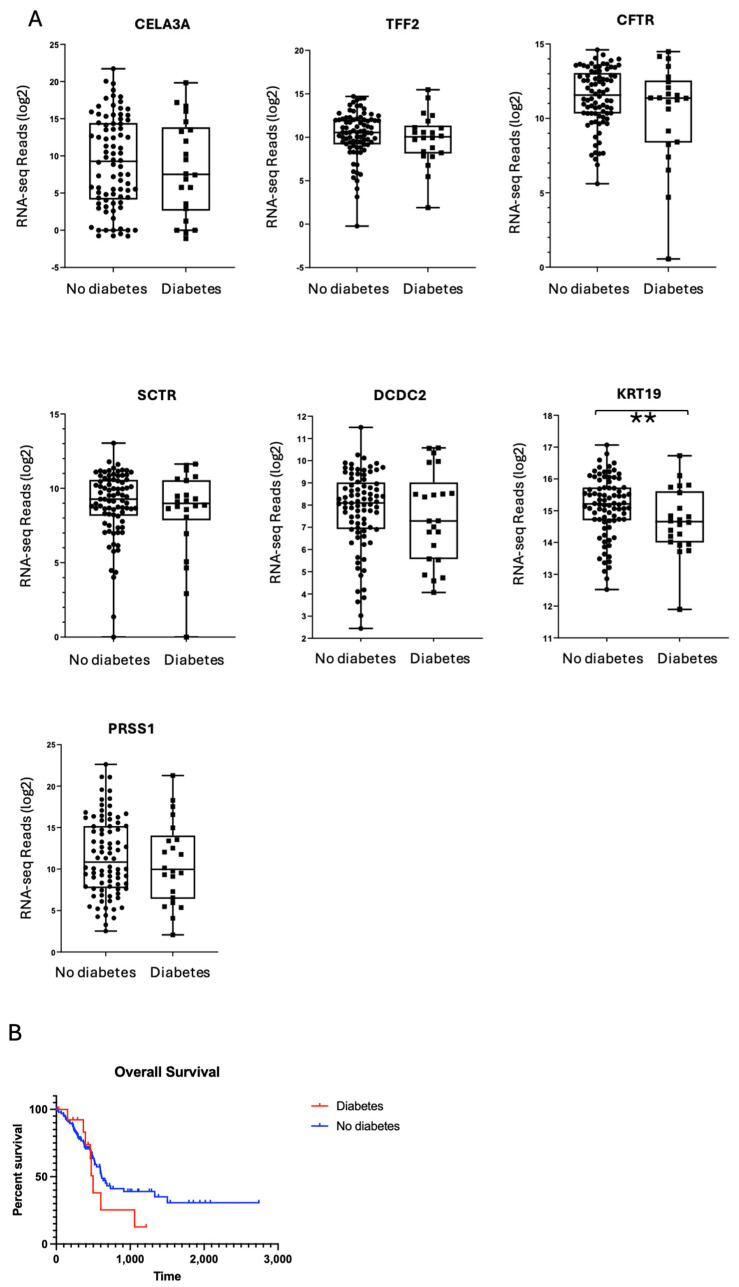
Gene expression analysis of exocrine markers across cohorts. (**A**) Genes associated with the exocrine tissue had similar expression across cohorts (*p* > 0.05) except KRT19, which was significantly reduced in the diabetic cohort (** *p* < 0.05). (**B**) Comparison of clinical outcomes for PDAC with diabetes cohort versus no diabetes cohort. Clinical data were downloaded from the Firehose server and plotted as a Kaplan–Meier curve based on presence or absence of diabetes. Mean survival for the diabetes cohort = 498 days, no diabetes cohort = 614 days. Time = days, *p* value = 0.3.

We next assessed whether PDAC-associated diabetes might alter exocrine cell markers or lead to greater fibrotic remodelling. Gene markers of ductal cells (e.g., Chymotrypsin-Like Elastase Family Member 3A (CELA3A), Trefoil Factor 2 (TFF2), Cystic Fibrosis Transmembrane Conductance Regulator (CFTR), Secretin Receptor (SCTR), Doublecortin Domain Containing 2 (DCDC2)) and acinar cells (Protease, Serine 1 (PRSS1)) were similarly expressed in diabetic and non-diabetic donors (Figure 5). Likewise, established markers of fibrosis (collagens, elastin, fibronectin, fibrin) were not differentially expressed between the two groups.

Interestingly, the one notable exception was Keratin 19 (KRT19), a ductal epithelial marker, which showed reduced expression in PDAC-associated diabetes relative to the non-diabetic PDAC cohort. Previous work linked high KRT19 expression in PDAC tumours to poorer survival outcomes [21]. However, in our dataset, Kaplan–Meier analysis revealed no significant difference in overall survival between diabetic and non-diabetic groups (Figure 4B). Thus, while KRT19 expression was unexpectedly lower in diabetic PDAC samples, this reduction did not confer a survival benefit or a distinct clinical trajectory in our cohort.

### 2.5. PDAC-Associated Diabetes Does Not Represent a Distinct Immunogenic Subtype

**Figure 6:**
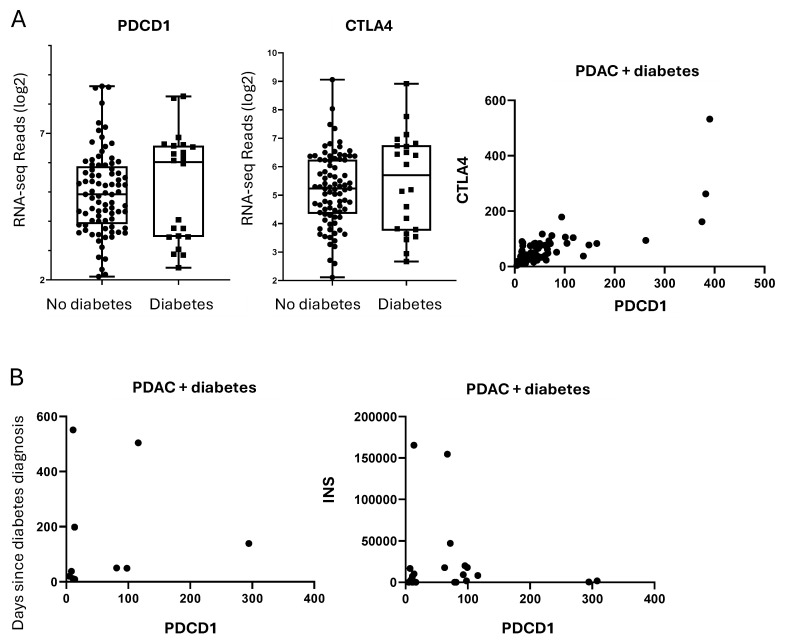
Gene expression analysis for genetic markers of immunogenic subtype. (**A**) PDCD1 expression had a binary pattern in the diabetic group (*p* > 0.05). Correlation analysis between PDCD1 and CTLA4 expression in the diabetic cohort was significant (r = 0.9, *p* < 0.001) (**B**). No correlation was observed between PDCD1 and duration of diabetes (r = 0.4), similarly between PDCD1 and INS in the diabetic cohort (r = 0.09).

We profiled the expression of PDCD1 (PD-1) and CTLA4, two immune checkpoint genes previously implicated as defining immunogenic subsets of PDAC-associated diabetes [8]. Although a clear binary (high vs. low) pattern in PDCD1 expression emerged across the full PDAC cohort, neither PDCD1 nor CTLA4 was selectively elevated in the diabetic group (Figure 6). These findings indicate that PDAC-associated diabetes does not uniformly adopt a “checkpoint-high” immunophenotype. Additionally, PDCD1 did not correlate with time since diabetes diagnosis, INS, or GCG, suggesting a lack of a direct link between islet hormone depletion and PD-1–mediated immune modulation.

To further substantiate these observations, we examined the relationships between PDCD1 and Cluster of Differentiation (CD) markers of various leukocyte subsets (e.g., CD8, CD45, CD72, CD38; Supplementary Figure S2). As expected, PDCD1 positively correlated with certain immune cell–associated transcripts, but these correlations did not distinguish the diabetic subset from non-diabetic samples. Hence, our data collectively suggest that PDAC-related diabetes is not characterized by a unique checkpoint profile.

### 2.6. Immune Cell Infiltration Shows No Significant Differences in PDAC with Diabetes

**Figure 7:**
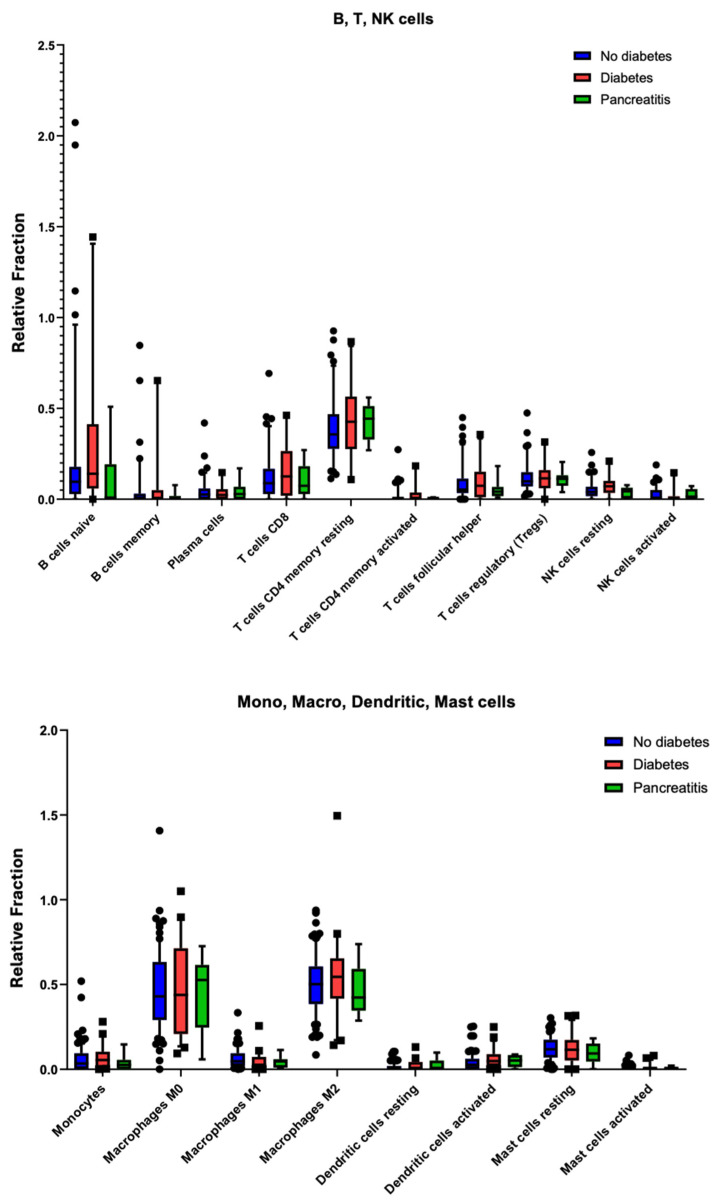
Comparison of relative leukocyte populations in the tumour microenvironment of PDAC (with and without diabetes) and pancreatitis as determined by CIBERSORT. No statistical significance was observed but the PDAC with diabetes group had higher levels of CD4 T cell markers. Round dots indicate no diabetes, and squares indicate with diabetes.

As checkpoint gene expression alone may not capture the entire immune landscape, we used the CIBERSORT deconvolution algorithm [22] to estimate the relative proportions of 22 distinct leukocyte populations in the tumour microenvironment (Figure 7). Consistent with the checkpoint analyses, no statistically significant differences were observed in the frequency of B cells, T cells (CD4^+^, CD8^+^, regulatory, or memory subsets), natural killer (NK) cells, monocytes, macrophages (M0, M1, or M2), dendritic cells, or mast cells when comparing PDAC donors with and without diabetes.

We extended these analyses to a small pancreatitis subset (green bars), which similarly showed no marked deviations in immune cell composition relative to PDAC samples (diabetic or non-diabetic). Although we noted a trend toward increased memory T-cell populations in the diabetes and pancreatitis groups, these differences were not statistically significant. Together, these results argue against the hypothesis that PDAC-associated diabetes constitutes a distinct inflammatory or immunogenic subtype within pancreatic cancer.

## 3. Discussion

Long-standing diabetes confers an elevated risk of various malignancies—including pancreatic cancer—underscoring the connection between metabolic dysregulation and tumourigenesis [7,23]. Intriguingly, de novo or rapidly worsening hyperglycemia in older adults may itself herald the onset of PDAC, prompting increased efforts to characterize the mechanisms underlying PDAC-associated diabetes [24]. Identifying and understanding these mechanisms could facilitate earlier PDAC detection, and surgical intervention or targeted therapies may yield improved outcomes.

In the present study, we provide evidence that PDAC-associated diabetes is distinguished by a global downregulation of key islet hormones (INS, GCG) as well as β-cell-enriched transcription factors such as MAFA and PAX4, and hormone receptors such as GCGR, SSTR3 and SSTR5. This pattern differs markedly from both type 1 and type 2 diabetes, wherein β-cell function declines but α-cell function is often maintained or upregulated—resulting in hyperglucagonemia. By contrast, PDAC-associated diabetes appears to affect all major endocrine cell lineages within the islets, suggesting that tumour-induced perturbations override conventional diabetic pathways. The reduction in GCGR expression would disrupt the positive reinforcement of insulin release from β-cells by glucagon within the islet, while the downregulation of SSTRTs −3 and −5 would prevent the fine negative control of both glucagon and insulin release by somatostatin [25], although the expression of SST itself was not observed to have significantly changed. The absence of correlations between diabetes duration and islet hormone gene expression further supports a local, tumour-driven aetiology that disrupts the paracrine homeostasis of the islets rather than a more common metabolic or autoimmune pathology. Clinically, these observations echo earlier findings that PDAC resection can mitigate new-onset diabetes in some patients [5,26], underscoring the direct influence of tumour biology on glycemic status.

One observation revealed by our histological analyses was pronounced thickening of the basement membrane surrounding islets in PDAC tissue. Although basement-membrane changes are well documented in microvascular complications of long-standing diabetes—such as nephropathy and retinopathy—reports of marked peri-islet basement-membrane thickening are sparse, even in advanced type 1 or type 2 diabetes. By examining the TCGA database and comparing samples from the University of Exeter’s Diabetes Archival Database, we found that basement-membrane thickening around islets was noticeable only in PDAC-affected tissue. While confounding factors such as hyperglycemia or local inflammation could contribute to this phenomenon, the extensive desmoplastic response characteristic of PDAC is likely central to such pronounced matrix remodelling. Thickening of the islet basement membrane may not be reflected in genetic data due to its primarily structural nature, which is driven by post-transcriptional modifications, protein accumulation, or altered extracellular matrix remodeling rather than significant changes in gene expression. However, altered gene expression was found for some matrix-modifying genes such as ADAM23. Furthermore, the islet basement membrane constitutes a very small fraction of the pancreatic tissue, and its signal is likely diluted in bulk RNA sequencing, which captures the average transcriptional activity of the entire pancreas. This limitation highlights the need for proteomic analyses or spatially resolved techniques, such as single-cell RNA sequencing or immunohistochemistry, to better understand the molecular mechanisms underlying basement membrane thickening in diabetes.

These findings raise the possibility that peri-islet basement-membrane expansion may be a unique histopathological marker of PDAC, potentially reflecting fibroinflammatory crosstalk between cancer cells, cancer-associated fibroblasts (CAFs), and the surrounding extracellular matrix (ECM). The basement membrane plays a critical role in islet function by regulating the exchange of nutrients, hormones, and signalling molecules between endocrine cells, blood vessels, and the exocrine pancreas. Thickening of the basement membrane could impair vascular flow within the islets and reduce nutrient and oxygen diffusion to β-cells, leading to metabolic stress and endocrine dysfunction [27]. Islets are particularly sensitive to changes in vascular efficiency since blood enters human islets through as little as single arterioles per islet that penetrates the peri-islet basement membrane [28]. A thickening of the basement membrane in PDAC may severely limit the islet arteriolar vascular flow with negative pan-endocrine cell consequences. CAFs represent a heterogeneous population of activated fibroblasts. These cells secrete extracellular matrix proteins, growth factors, and cytokines that not only support tumour growth but also potentially disrupt islet architecture and function. Recent evidence suggests that CAFs can secrete inflammatory mediators such as Interleukin (IL)-6, IL-1β, and Tumour necrosis factor -alpha (TNF-α) [29], which are known to impair insulin secretion and promote β-cell apoptosis through paracrine signaling. This is consistent with a model where tumour–islet crosstalk, mediated by CAFs and the desmoplastic stroma, affects multiple endocrine cell types simultaneously. The bidirectional relationship between CAFs and islet cells may create a feed-forward loop that accelerates both diabetes development and tumour progression. CAF-derived factors may impair islet function, leading to hyperglycemia, which in turn can promote further CAF activation and tumour growth. A more detailed investigation into how CAFs communicate with endocrine cells at the molecular levels could uncover therapeutic targets for interrupting or managing the diabetes-cancer cycle in PDAC.

Recent work has implicated β-cells as active drivers of PDAC progression rather than as passive responders to tumour-induced metabolic dysregulation. Garcia et al. [30] demonstrated that β-cell ablation impedes PDAC development, suggesting a direct role for endocrine–exocrine signalling in tumourigenesis. It is possible that early β-cell hyperactivity contributes to tumour initiation, whereas later-stage PDAC progression involves β-cell depletion and endocrine failure. This progression may explain why diabetes often emerges following tumour growth—initially, β-cells may be overstimulated and promote tumourigenesis through endocrine–exocrine signalling, but as the tumour expands and disrupts normal pancreatic architecture, β-cell function declines, leading to insulin insufficiency and diabetes onset.

PDAC is characterized by dense fibrotic stroma, typified by excessive collagen deposition and a robust desmoplastic reaction [31,32]. Our findings are consistent with this well-documented stromal expansion, as we observed substantial collagen deposition both within and around islets, often displacing normal acinar structures. Despite this extensive fibrosis, no distinct immunophenotype characterized by immune-checkpoint profiles or specific leukocyte populations emerged in PDAC-associated diabetes relative to PDAC without diabetes. These data suggest that while the tumour microenvironment in PDAC can be profoundly immunosuppressive and structurally prohibitive, it may not uniquely reorganize immune cell populations in those patients presenting with diabetes.

It is increasingly appreciated that the pancreas is characterized by extensive regional heterogeneity, with islet size, composition, and vascularization varying across different regions [33]. This variation likely contributes to the highly variable metabolic presentation of PDAC-associated diabetes. Even within a single patient, some islets may remain relatively intact while others become severely compromised by local tumour growth and fibrosis. The heterogeneous nature of islet remodelling may help explain why glycemic status improves in certain individuals following surgical resection of PDAC [5,26].

From a clinical vantage point, these findings highlight the importance of vigilant metabolic monitoring in patients over 50 years of age who exhibit sudden-onset diabetes or unexplained deteriorations in glycemic control. Although current screening protocols for pancreatic cancer in the general population remain challenging and costly, identifying those at heightened risk via new-onset diabetes may allow for earlier imaging, tumour detection, and intervention.

Taken together, our data reveal that PDAC-associated diabetes arises through a convergent mechanism involving broad endocrine disruption and potentially unique architectural changes in the islet microenvironment—including a thickening of the islet basement membrane. These observations argue for a model in which local tumour–islet crosstalk, dense fibrotic stroma, and altered ECM contribute to the rapid decline in insulin, glucagon, and somatostatin production. Future multidisciplinary studies combining transcriptomics, spatial proteomics, advanced imaging, and robust clinical phenotyping are needed to confirm and expand upon these findings.

Our findings suggest several potential therapeutic approaches for PDAC-associated diabetes. Metabolic interventions addressing multiple hormone deficiencies may better manage the global endocrine disruption we observed. Additionally, monitoring endocrine marker downregulation in high-risk individuals could serve as an early detection strategy for PDAC when surgical intervention remains viable. This study provides the first comprehensive integration of curated TCGA transcriptomic and histopathological evidence. Our study challenges the view held by some in the field that PDAC-associated diabetes merely represents a Type 2 phenotype, providing evidence that it should be analyzed and treated as its own distinct class of diabetes with unique pathophysiological mechanisms. Our novel identification of basement membrane thickening as a potential histopathological marker of PDAC distinguishes this work from previous studies and offers new insights into the mechanisms of tumour-induced endocrine dysfunction. Future research should elucidate the specific molecular pathways underlying tumour–islet crosstalk and endocrine disruption to identify novel therapeutic targets and develop more effective treatment strategies for this distinct form of diabetes. Understanding these complex interactions may ultimately improve both glycemic control and survival outcomes in this notoriously lethal malignancy.

## 4. Materials and Methods

### 4.1. Patient Data Collection

Patient data were retrieved from The Cancer Genome Atlas (TCGA), which provides a comprehensive, multidimensional map of genomic alterations in PDAC. Level 3 RNA sequencing data, normalized using the RNA-seq by Expectation Maximization (RSEM) method, were extracted from the Illumina HiSeq platform (https://gdac.broadinstitute.org/) (accessed on 3 June 2023). The TCGA/PanCancer Atlas PDAC cohort was downloaded from the Broad Genome Data Analysis Centers Firehose server (https://gdac.broadinstitute.org/) (accessed on 3 June 2023). The gene-level Firehose dataset was utilized for all transcriptomic analyses. Written informed consent was obtained from the patients (or their families) as part of the TCGA project.

To ensure the accuracy of the dataset, patient cohorts underwent rigorous curation based on quality control standards outlined by Peran et al. [34] (Figure 1). Samples previously identified as misclassified or of uncertain diagnosis were removed. Additionally, patients with a recorded history of chronic pancreatitis—or for whom such history was unknown—were excluded from the study, given that pancreatitis is a significant confounder in diabetes risk, contributing to an estimated 25–75% of diabetes cases depending on disease aetiology [35].

The final study cohort included 114 patients with PDAC, of whom 30 had a documented diagnosis of PDAC-associated diabetes, while 84 had PDAC without a recorded diabetes diagnosis. The initial dataset comprised 183 patients from TCGA, but non-PDAC malignancies were excluded following rigorous verification using clinical data and pathology reports (Figure 1).

Within the PDAC-associated diabetes group, 30 patients were initially identified; however, 8 patients were excluded due to a history of pancreatitis, which can independently contribute to diabetes development. The mean duration of diabetes at the time of data collection was 967 days, with 7 patients diagnosed within 150 days of tumour resection. The mean ages of the diabetes and non-diabetes cohorts were 66 and 65 years, respectively.

The dataset utilized comprehensive clinical metadata encompassing patient demographics (age, gender, race), clinical outcomes, tumour pathology, treatment modalities, and lifestyle factors such as smoking status. Gene expression data were derived from RSEM-normalized RNA sequencing, ensuring standardized processing and comparability across the TCGA PDAC cohort.

Because the TCGA dataset does not include histological samples for all patients—and robust non-PDAC control tissues are absent—additional pancreatic samples were obtained from the University of Exeter’s Diabetes Archival Database (accessed via https://www.pancreatlas.org/datasets) (accessed on 10 July 2024), a large biorepository encompassing donors with no known pancreatic pathology as well as those with various diabetes aetiologies. These specimens served as a reference for baseline islet architecture and typical basement-membrane thickness.

### 4.2. Tissue Analysis

Histological evaluation was performed on tissue samples retrieved from TCGA. Whole-slide imaging data were accessed through the Cancer Digital Slide Archive (http://cancer.digitalslidearchive.net) (accessed on 10 July 2024). To ensure consistency, tissue selection criteria included histopathological confirmation of PDAC without confounding pancreatic lesions. Due to the multi-centre nature of TCGA sample collection, fixation protocols were not standardized, necessitating careful histological review to minimize artefactual inconsistencies. Where available, confirmatory assessments of islet integrity, fibrosis, and cellular composition were conducted on digital histology slides, with fibrosis scored using a semi-quantitative grading system across multiple regions per sample as described by Korpela et al. [36].

Automated software-based quantification of islets was not feasible due to several intrinsic limitations of the TCGA dataset. First, TCGA tissue samples were obtained from different anatomical regions of the pancreas without a standardized selection process for islet inclusion. As PDAC primarily affects the exocrine pancreas, many TCGA sections contained minimal or no visible islets, making systematic quantification unreliable. Additionally, the number of islets per histological section does not necessarily reflect overall islet abundance in the pancreas, as islets are distributed heterogeneously and their presence in sampled tissue is incidental rather than systematically curated. Second, automated detection and segmentation of islets in TCGA images were hindered by variability in staining protocols and image quality across contributing institutions. Differences in haematoxylin and eosin (H&E) staining intensity, fixation artifacts, and sectioning inconsistencies introduce challenges for machine learning-based image analysis tools, which require standardized training datasets for reliable performance. Furthermore, islet morphology can be significantly altered in PDAC due to fibrosis and tumour infiltration, complicating automated recognition. Given these limitations, a manual, semi-quantitative scoring approach was adopted to assess islet presence, fibrosis, and structural integrity across available samples.

### 4.3. Differential Gene Expression and Volcano Plot Analysis

RNA-sequencing data from TCGA were processed to identify differentially expressed genes between PDAC patients with and without diabetes. Gene expression levels were normalized using the RNA-seq by Expectation Maximization (RSEM) method. Differential expression analysis was performed using limma-voom, and statistical significance was determined using Student’s *t*-test with an adjusted *p*-value threshold of <0.05. Log_2_ fold change values were calculated to assess the magnitude of differential expression. A volcano plot was generated to visualize the relationship between statistical significance (log_10_ *p*-value) and fold change (log_2_FC) using Matplotlib (version 3.3.4) and Seaborn (version 0.11.1) in Python (version 3.8). Genes above the significance threshold (dashed red line) were considered significantly differentially expressed.

### 4.4. Statistical Analysis

All statistical analyses were performed using GraphPad Prism v7.0 (GraphPad Software, Inc., San Diego, CA, USA) unless otherwise specified. Boxplot comparisons of gene expression levels were generated, with centre lines indicating medians, box limits denoting the interquartile range (IQR), and whiskers extending to the minimum and maximum values. Statistical significance for pairwise comparisons was determined using the Mann–Whitney U test (one-tailed, *p* < 0.05). Correlation analyses were conducted using Spearman’s Rho, with statistical significance set at *p* < 0.05. To address multiple hypothesis testing concerns, false discovery rate (FDR) adjustments were applied where necessary using the Benjamini–Hochberg method.

### 4.5. CIBERSORT Immune Deconvolution

CIBERSORT [22] was utilized to estimate the relative proportions of 22 distinct immune cell phenotypes based on bulk RNA sequencing data from TCGA PDAC samples. This methodology employs a 547-gene signature matrix to deconvolute immune cell subtypes—including T-cell subsets, B cells, natural killer (NK) cells, and myeloid populations. Immune deconvolution was performed on pre-processed RSEM-normalized TCGA expression data. The accuracy of immune cell fraction estimates was evaluated using CIBERSORT’s permutation-based statistical framework, with significance determined at an FDR threshold of <0.05. To ensure consistency with our targeted immune analysis, we compared CIBERSORT-derived immune cell distributions with key marker gene expression data analysed in Supplementary Figure S2, providing cross-validation of immune classification results.

## Data Availability

The data supporting the results of this study are publicly available. Gene expression and clinical data were obtained from The Cancer Genome Atlas (TCGA), accessible at https://portal.gdc.cancer.gov/ (accessed on 3 June 2023). Histological images were retrieved from the Cancer Digital Slide Archive, available at http://cancer.digitalslidearchive.net (accessed on 10 July 2024). In addition, pancreatic tissue samples used as references were obtained from the University of Exeter’s Diabetes Archival Database, which can be accessed at https://www.pancreatlas.org/datasets (accessed on 10 July 2024). No new data were generated in this study. Researchers are encouraged to consult the respective repositories for further details on data access and usage in accordance with their policies.

## Supplementary Materials

The following supporting information can be downloaded at: https://www.mdpi.com/article/10.3390/ijms26073191/s1.

## Abbreviations

ADAM32	Disintegrin And Metalloproteinase Domain-Containing Protein 32
ARNT	Aryl Hydrocarbon Receptor Nuclear Translocator
ARX	Aristaless Related Homeobox
BAIAP3	BAI1 Associated Protein 3
BCL2L11	BCL2 Like 11
BM	Basement Membrane
CAFs	Cancer-Associated Fibroblasts
CD38	Cluster of Differentiation 38
CD45	Cluster of Differentiation 45
CD72	Cluster of Differentiation 72
CD8	Cluster of Differentiation 8
CELA3A	Chymotrypsin-Like Elastase Family Member 3A
CHGA	Chromogranin A
CIBERSORT	Cell-type Identification By Estimating Relative Subsets Of RNA Transcripts
CFTR	Cystic Fibrosis Transmembrane Conductance Regulator
CRTAP	Cartilage Associated Protein
CTLA-4 (CTLA4)	Cytotoxic T-Lymphocyte Associated Protein 4
CBCL5	C-C Motif Chemokine Ligand 5
DCDC2	Doublecortin Domain Containing 2
DOC2A	Double C2 Domain Alpha
ECM	Extracellular Matrix
FGFR4	Fibroblast Growth Factor Receptor 4
FDR	False Discovery Rate
FOXP1	Forkhead Box P1
GCG	Glucagon
GCK	Glucokinase
H&E	Hematoxylin and Eosin
HGFAC	Hepatocyte Growth Factor Activator
HOXB	Homeobox B Cluster
INS	Insulin
KCNK16	Potassium Two Pore Domain Channel Subfamily K Member 16
KRT19	Keratin 19
MAFA	MAF BZIP Transcription Factor A
MAFB	MAF BZIP Transcription Factor B
NANOG	Nanog Homeobox
NEUROD1	Neuronal Differentiation 1
NEUROD4	Neuronal Differentiation 4
NKX1-2	NK1 Homeobox 2
PDAC	Pancreatic Ductal Adenocarcinoma
PDGFA	Platelet Derived Growth Factor Subunit A
PD-1 (PDCD1)	Programmed Cell Death Protein 1
PD-L1	Programmed Death-Ligand 1
PAX4	Paired Box 4
PRSS1	Protease, Serine 1
RIMS2	Regulating Synaptic Membrane Exocytosis 2
RBPJ	Recombination Signal Binding Protein For Immunoglobulin Kappa J Region
RSEM	RNA-seq by Expectation Maximization
SCTR	Secretin Receptor
SMAD3	SMAD Family Member 3
SST	Somatostatin
SSTR	Somatostatin receptor
TFF2	Trefoil Factor 2
TCGA	The Cancer Genome Atlas
WSCD2	WSC Domain Containing 2
