# Supplementary material for: Gene Dysregulation and Islet Changes in PDAC-Associated Type 3c Diabetes
Source: Int J Mol Sci. 2025 Mar 29;26(7):3191. doi: 10.3390/ijms26073191 (PMC11988973; doi:10.3390/ijms26073191)
Supplement: Supplementary file 1 [file ijms-26-03191-s001.zip › ijms-3517873-supplementary.pdf]

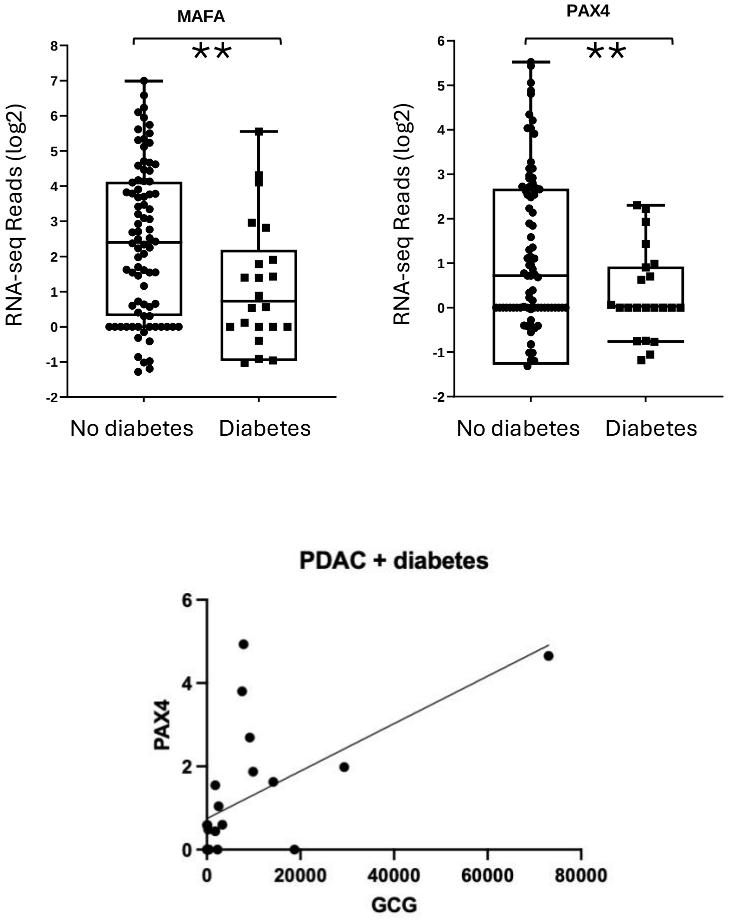

Supplementary Figure S1. Markers of endocrine progenitor cells reduced in diabetic cohort. Gene expression of MAFA and PAX4 was significantly reduced (***p*<0.05) in the diabetic cohort. PAX4 and GCG expression had a significant positive correlation (r = 0.65).

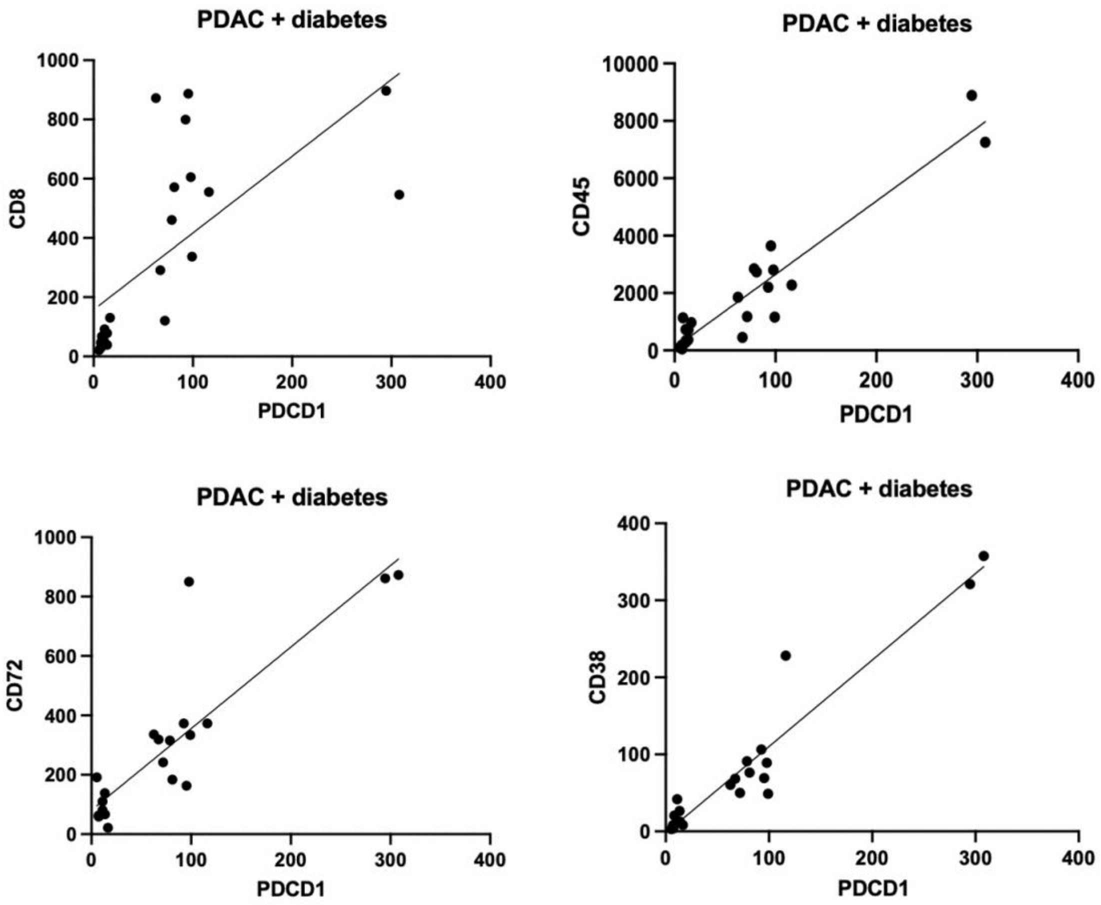

Supplementary Figure S2. Correlation analysis between PDCD1 and markers of immune cells and checkpoint inhibitors. Expression of PDCD1 was found to significantly correlate with immune markers CD8 (T cells) (r = 0.8), CD45 (hematopoietic cells) (r = 0.87), CD72 (B cells) (r = 0.8) and CD38 (immune cell activation) (r = 0.9).

Supplementary Table S1

Genes associated with islet cell function significantly lower in pancreas expression in PDAC with diabetes vs. no diabetes

Transcription factors

Gene(s)	Notes / Function
**HoxB1, HoxB3, HoxB6**	Homeobox genes associated with islet endocrine cell lineage plasticity
**MafA**	Transcription factor defining β-cell maturity
**NeuroD1, NeuroD4**	Neurogenic Differentiation Factors; involved in differentiation of endocrine cell lineages
**Nanog**	Regulator of endocrine cell lineage commitment
**Pax4**	Transcriptional regulator of β-cell lineage commitment
**NKX1-2**	Transcriptional regulator of β-cell lineage commitment
**HNF4G**	Oncogene for islet endocrine progenitor cells
**RFX6**	Essential transcription factor for endocrine pancreas development. Controls insulin gene transcription and maintains gene expression and function of α-cells.

Hormones and receptors

**INS**	Insulin
**GCG**	Proglucagon
**GCGR**	Glucagon receptor
**CRH**	Corticotrophin-releasing hormone; expressed by β-cells
**IAPP**	Encodes amylin (islet amyloid polypeptide) that can impair β-cell function
**GHSR**	Ghrelin receptor; regulates somatostatin secretion
**GPR39, GPR44, GPR81, GPR119, GPR142**	GPCRs mediating ligand-initiated endocrine functions
**SSTR3, SSTR5**	Somatostatin receptors on α- and β-cells regulating hormone release
**FGFR4**	Fibroblast growth factor receptor 4; mediates proliferative effects of FGF
**PDGFA**	Platelet-derived growth factor subunit-A; involved in islet cell proliferation
**HGFAC**	Activates hepatocyte growth factor, promoting β-cell proliferation

Islet intracellular pathways

**GCK**	Glucokinase, acting as a “glucose sensor” in β-cells
**G6PC2**	Glucose-6-phosphatase catalytic subunit 2; modulates glucose-stimulated insulin secretion

**C15orf58**	GDP-D-glucose phosphorylase 1; involved in β-cell glucose metabolism
**PTPRN**	Protein tyrosine phosphatase receptor type N (IA-2); critical for insulin secretion
**BCL2A1**	Regulator of endocrine cell apoptosis
**KCNK16**	Potassium channel subfamily K member 16; enhances glucose-stimulated insulin secretion
**DOC2A**	Double C2-like domains alpha; involved in insulin granule exocytosis
**RIMS2**	Regulating Synaptic Membrane Exocytosis 2; facilitates insulin granule exocytosis
**BAIAP3**	BAI1-associated protein 3; plays a role in dense-core hormone vesicle exocytosis
**CHGA**	Chromogranin A. Component of α-, β-, and δ-cells secretory granules
**ABCC8**	Encodes the SUR1 subunit of the β-cell K-ATP channel; crucial for glucose-stimulated insulin secretion
**KCNJ11**	Encodes Kir6.2, the pore-forming subunit of the β-cell K-ATP channel; essential for insulin secretion
**PCSK2**	Proprotein convertase 2 (PC2); involved in processing proinsulin to insulin and proglucagon to glucagon
**TRPM3**	Transient receptor potential cation channel M3; modulates Ca^{2+} influx and insulin secretion in β-cells

Extracellular matrix

**SMAD3**	Regulator of TGF-β receptor signaling and fibrosis
**ADAM32**	Metalloproteinase involved in extracellular matrix remodeling in cancer
**CXCL5**	Chemokine promoting remodeling of connective tissues

Genes associated with islet cell function significantly higher in pancreas expression in PDAC with diabetes vs. no diabetes

Transcription factors

**Gene(s)**	**Notes / Function**
**RBPJ**	Core transcription factor of the Notch pathway; regulates islet progenitor differentiation.
**ARNT**	Aryl hydrocarbon receptor nuclear translocator (also known as HIF1β); required for normal β-cell function. Known to be involved in stress response pathways.
**FOXP1**	Forkhead box transcription factor. Known to be involved in stress response pathways.

Islet intracellular pathways

**TMEM110**	Involved in store-operated Ca^{2+} entry (STIM-activating enhancer); crucial for insulin granule exocytosis in β-cells.

**BCL2L11**	Also known as BIM; a pro-apoptotic factor that may promote β-cell death under stress.
**STIM1**	Transmembrane protein that mediates Ca^{2+} influx into endocrine cells
**RGS10**	Regulator of G-protein signalling; can modulate GPCR signalling relevant to insulin/glucagon pathways.

Extracellular matrix

**CRTAP**	Collagen Type 1 formation
